# Evaluating the impact of local alcohol licensing decisions on outcomes for the community: a systematic review

**DOI:** 10.1136/bmjph-2023-000533

**Published:** 2024-01-18

**Authors:** Lindsay Blank, Emma Hock, Mark Clowes, Marie Rogerson, Elizabeth Goyder

**Affiliations:** 1 Sheffield Centre for Health and Related Research (SCHARR), School of Medicine and Population Health, The University of Sheffield, Sheffield, UK

**Keywords:** public health, community health, preventive medicine, public health practice

## Abstract

**Background:**

International evidence reviews suggest that reducing the availability of alcohol positively impacts both levels of alcohol consumption and associated harms. To understand the impact of recent changes to alcohol licensing and public health in the UK, this review aimed to identify and synthesise quantitative research evidence on the impact of local alcohol licensing decisions on the health and well-being of the community.

**Methods:**

We searched peer-reviewed articles and grey literature for UK studies. We extracted and tabulated key data from the included papers and appraised study quality. We included topic expert and public consultation to confirm the scope of the evidence synthesis and suggest evidence for inclusion. We synthesised narratively and made recommendations based on our findings.

**Results:**

We identified a small volume (seven papers) of evidence regarding the health (and related) impacts of local alcohol licensing decision undertaken in the UK local authorities. The evidence we identified did not demonstrate a consistent or sustained association between local interventions and health or crime outcomes downstream. This was despite relatively sophisticated study designs using a range of available data sources and some longer-term analysis.

**Conclusion:**

Given that the impacts of local licensing decisions are currently limited, greater regulatory powers are needed if local licensing interventions are to be an effective public health interventions to reduce alcohol-related harms.

**WHAT IS ALREADY KNOWN ON THIS TOPIC**

**WHAT THIS STUDY ADDS**

- The available evidence does not demonstrate a consistent or sustained association between local alcohol licensing decisions and health or crime outcomes.

**HOW THIS STUDY MIGHT AFFECT RESEARCH, PRACTICE OR POLICY**

- Greater regulatory powers are needed locally for licensing interventions to have the potential to reduce alcohol-related harms.

## Introduction

 The consumption of alcohol is a substantial contributor to ill health internationally^1^ and the biggest risk factor attributable to early mortality and morbidity for those aged 15–49 years in the UK.^2^ In 2020, there were 8974 deaths from alcohol-specific causes registered in the UK; the highest year-on-year increase since the data time series began in 2001.^3^

In the UK, there are three different licensing regimens for the sale of alcohol. Revisions to the alcohol licensing act in 2003 in England and Wales established a ‘single integrated scheme for licensing premises used to sell or supply alcohol’.^4^ Licensing objectives were established to inform decisions to grant, amend or refuse licence applications. These objectives are: preventing crime and disorder, promoting public safety, preventing public nuisance and protecting children from harm. With the subsequent transfer of many public health functions from the National Health Service (NHS) to local government in England and Wales, local public health teams obtained a statutory role in the provision of alcohol licences.^5^ As a result of these changes, the commitment of public health resources to influence licensing decisions has increased.^6^ Since 2005, there is also an additional licensing objective to protect and improve public health in Scotland (but no statutory role for public health teams here or in Northern Ireland).

International evidence reviews suggest that reducing availability of alcohol has a positive effect on both levels of alcohol consumption and associated harms.^7^,^13^ Although there is compelling evidence to suggest that reducing availability is an effective and cost-effective approach to reducing alcohol consumption, harm and healthcare costs,^1^ the mechanisms which underlie these relationships are unclear.^10^ There is also an evidence gap for the UK-specific evidence reviews based on comparative studies, rather than descriptive and discursive evidence.

To understand the impact of changes to licensing decisions made locally, this review aimed to identify and synthesise quantitative research evidence on the impact of local authority alcohol licensing decisions on the health and well-being of the community.

## Review question: what is the impact of local alcohol licensing decisions on health and well-being outcomes for the community?

### Methods

The aim of this review was to identify, appraise and synthesise quantitative evidence (intervention studies and quantitative observational studies) from published research, policy documents and grey literature on the relationship between local alcohol licensing decisions and the health and well-being of communities. We followed the Cochrane Rapid Review Guidelines.^14^ We included topic expert and public consultation to refine the inclusion criteria and confirm the scope of the evidence synthesis. The protocol of our review is available online and is registered with Prospero (CRD42022362917).

### Inclusion criteria

Population: people living in the UK in an area affected by an alcohol licensing decision.

Intervention: change in alcohol licensing process or in alcohol licensing decisions made locally including off licensing and temporary licences.

Outcomes: all quantitatively measured outcomes of the health and well-being of the local population including impact on health inequalities.

Comparators: control areas or before and after analysis depending on study designs.

### Searching

#### Database searching

Searches were conducted in November 2022. A search strategy comprising subject headings and free-text terms was developed in MEDLINE before being adapted for the other databases including Embase, Web of Science, Applied Social Sciences Index and Abstracts, PsycINFO and the International Bibliography of Social Sciences databases.

Where suitable search filters were available, searches were restricted to the UK. A sample search strategy, incorporating the National Institute for Health and Care Excellence (NICE) UK filter,^15^ is provided in online supplemental material.

#### Citation searching and additional methods

The following complementary approaches were used to identify additional evidence: consultation with local authority colleagues in Scotland, England, Wales and Northern Ireland, along with topic experts and public representatives; scrutiny of reference lists (of included papers and relevant systematic reviews); scrutiny of recent policy documents; citation searches of included papers; Web of Science search for related work by key authors (Niamh Fitzgerald and coauthors) including the terms alcohol* and licen*, and papers citing them.

To identify grey literature, we searched websites of organisations working in the alcohol field including: Alcohol Change; Alcohol Focus Scotland; Alcohol Health Alliance; Association of Directors of Public Health; Balance North-East; Department of Health and Social Care (.gov.uk); Institute of Alcohol Studies; Office for Health Improvement and Disparities; Public Health Scotland; Public Health Wales; Scottish Health Action on Alcohol Problems. Where no search functionality was available, we browsed the sites for relevant material following the guidance of Stansfield *et al.*^16^ Sites were initially browsed by the information specialist (MC) and results of possible relevance were selected for screening by the reviewers (LB and EH).

Sifting and study selection: Database search results were downloaded to a reference management system (EndNote) and screened against the inclusion criteria by one reviewer, with a 10% sample screened by a second reviewer. For each paper selected at the abstract level, the full paper was downloaded and screened against the inclusion criteria by two reviewers. Uncertainties were resolved by discussion among the review team.^14^

Grey literature searches and screening were tabulated (see online supplemental material).^16^ Titles were screened against the inclusion criteria and the full documents were downloaded to facilitate full text screening. Each source was considered for inclusion by one reviewer, with queries checked in discussion with the whole team. Reference lists of included studies and relevant reviews were screened for potentially relevance. The full text of potentially relevant references was downloaded and examined for relevance by one reviewer.

### Data extraction and synthesis

A data extraction form, based on those used by the team for similar topics, was piloted by two reviewers and revisions were discussed and agreed with the review team. Data were extracted and tabulated as follows: first author, year of study, location of study, study design, analysis methods, population, intervention type and description, control condition, outcome(s) assessed, duration of measure, length/dates of study, results (narrative), results (data), data sources, conclusion and study limitations. Data extraction was performed by one reviewer and checked for accuracy by a second reviewer. We synthesised the findings narratively.

### Quality appraisal

We assessed the quality of the published literature using a modified version of the Critical Appraisal Skills Programme checklist for cohort studies^17^ (see online supplemental material). Due to the study designs employed, we were not able to identify a single published checklist that would accurately reflect the quality of the studies. The quality of grey literature was appraised using the Authority, Accuracy, Coverage, Objectivity, Date, Significance (AACODS) checklist.^18^ Quality assessment was performed by one reviewer and checked for accuracy and consistency by a second reviewer.

### Patient and public involvement

Stakeholders and topic experts were invited to comment on the focus of the review and the appropriate definitions and scope of review questions and inclusion criteria. Further consultations were undertaken to gain feedback and advice on the identification of evidence sources, the interpretation and implications findings and dissemination to diverse audiences. We included stakeholders from England, Scotland, Wales and Northern Ireland as local alcohol licensing policies vary (see online supplemental material).

A public advisory group consisting of seven individuals from across the UK were recruited from the National Institute for Health and Care Research (NIHR) ‘People in Research’ website.^19^ They provided advisory input via videoconferencing during the initial stage of searching, and during the interpretation of the analysis and synthesis. They proved valuable for understanding local contexts and the impacts of decisions on communities. Further public consultation on the review findings was sought through local government stakeholders in the four UK home nations.

## Results

### Included studies

**Figure 1:**
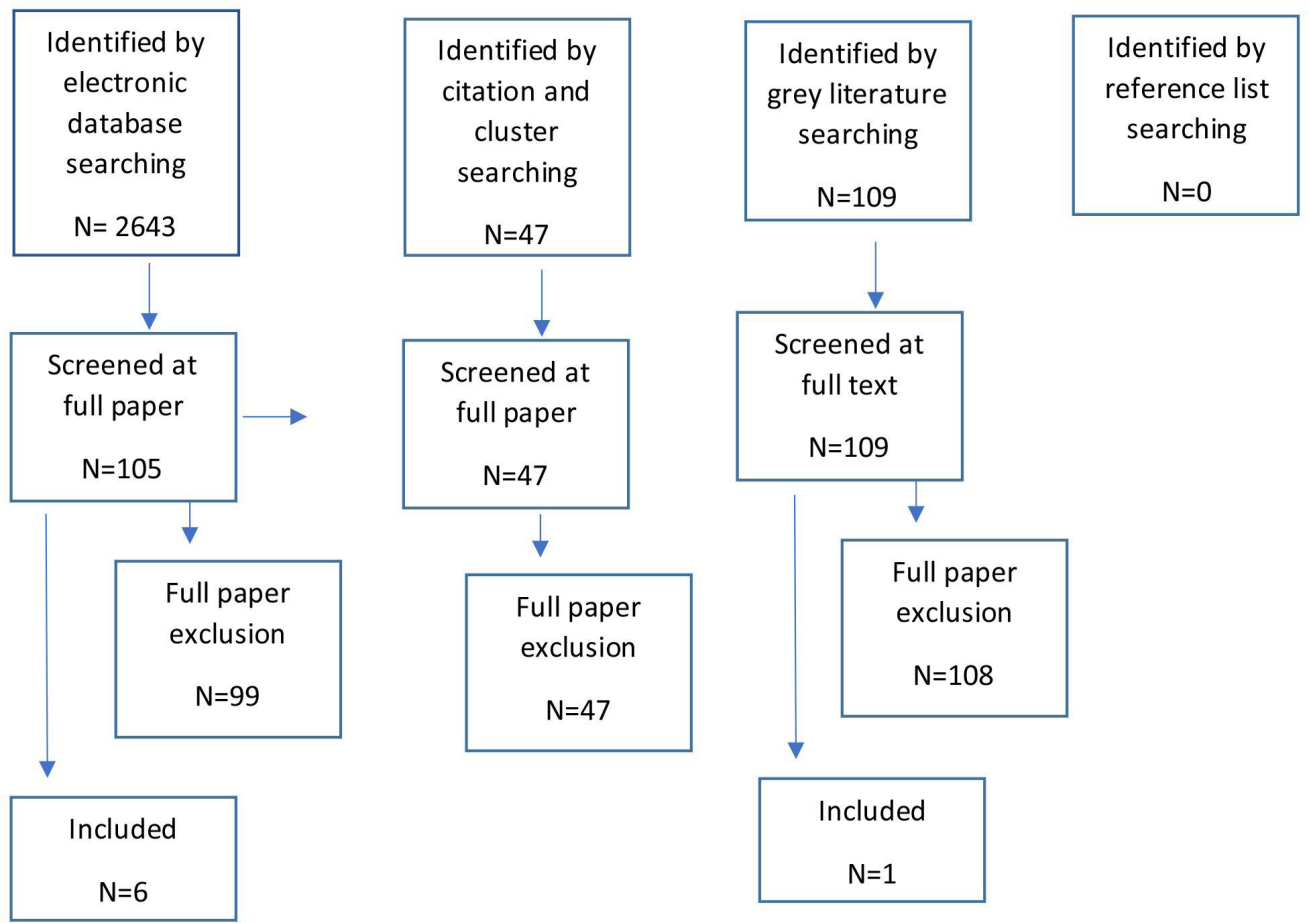
Flow chart of study identification and inclusion decisions.

Database searches generated 2690 unique references (figure 1). Citation searching identified 29 papers and cluster searching a further 18. A total of 105 articles were reviewed at the full paper stage. Six articles were found to meet our inclusion criteria. Reasons for exclusions at the full paper stage are provided in online supplemental material. Grey literature searches identified 109 potential sources of which only 1 was found to meet our inclusion criteria (see online supplemental material).

Within the confines of their study designs, the quality of the identified evidence was good. All studies address a clearly focused question with a sound research methodology and approach to data analysis. No studies were excluded based on quality. Quality appraisals of the included studies are presented in online supplemental material.

Ten papers were suggested by stakeholders during our consultation process. However, these were all duplications of papers identified in our searches. These consultations provided invaluable in understanding the variation in alcohol licensing approaches between the UK nations. Our public consultation provided local context around the impacts of alcohol licensing decisions on communities but did not contribute evidence directly to the review.

Therefore, six peer-reviewed papers and one grey report were included in our review. Although reporting on different studies with various funding sources published over an 8-year period between 2015 and 2022, these publications are linked by having authors in common across different studies and publications.

### Study designs

Studies consisted of interrupted time series of varying designs. Two of the papers contained a ‘before and after’ comparison within the same population.^20 21^ One paper reported on data which included matched controlled areas.^22^ Two papers used data which compared intervention areas to synthetic control areas.^23 24^ de Vocht *et al*^25^ included areas with diverse levels of activity over time, and de Vocht *et al*^26^ reported on the ‘control crime’ of financial fraud. The data analysed were collected over a period of 6–8 years between 2007 and 2019 overall. Studies included data collected in England only, with the exception of de Vocht *et al*,^25^ which also included data from Scotland.

### Interventions

Interventions were mostly implemented as part of Cumulative Impact Zones (CIZ): policies intended to regulate the availability of alcohol by controlling new alcohol outlets in a specific local area. Local authorities can use CIZs to control new alcohol outlets in areas of overprovision. Within a CIZ, new alcohol licence applicants must demonstrate how they will avoid threatening their licensing objectives.

In one case, data on the introduction of a single CIZ were collected in Islington, London.^20^ Pliakas *et al*^21^ evaluated a borough-wide guideline framework of closing times for businesses applying for new and variation alcohol licences. The new alcohol licensing policy included a comprehensive Cumulative Impact Policy enforced in seven (CIZs in one English local authority.^21^

In other papers, data were compared between CIZs where the degree of intensity of scrutiny/engagement in CIZ for each locality. In deVocht *et al*^22^ and de Vocht *et al,*^26^ this was coded as ‘passive’, low, medium or highly activity based on quartiles of the distribution (data on the presence of CIZ and on successful challenges of licences for new premises were recoded as present (1) or absent (0) and for each year added together to derive a three-level score indicating how active an area’s alcohol policy was).

Others included local areas where both a CIZ and further licensing enforcement were introduced.^23 25^ For the paper published in 2022,^25^ public health engagement in the licensing system (not an action or decision of the licensing system itself) was investigated. ‘The Public Health engagement In Alcohol Licensing (PHIAL) Measure’ was developed, which included activities in six categories: staffing; reviewing licence applications; responding to licence applications; data usage; influencing licensing stakeholders or policy; and public involvement. In de Vocht *et al*,^23^ intervention areas were local areas with no specified CIZ and no rejection of new licensing applications in 2007/2008, but with both implemented in 2011/2012 and thereafter. Controls were local areas with neither policy.

Further, de Vocht *et al*^24^ evaluated case study interventions in three English local areas. Interventions were described as nightclub and restaurant closures following review, and implementation of new local licensing guidance. These were not described as CIZ.

### Outcome measures reported and duration

Direct health outcomes included: hospital admissions related to alcohol misuse (alcohol-related hospital admissions^22 23^; emergency department admissions^26^ and hospital admissions for acute conditions related to alcohol^25^); ambulance call-outs (alcohol related^20 23^ or all call-out^24 25^); and alcohol-related mortality and alcohol-specific mortality^25^ (table 1.). Crime outcomes relating to well-being and community safety were reported including violent and sexual crimes^23 25 26^; antisocial behaviour,^23^ crime^21^ and alcohol-related crime.^24^ de Vocht *et al* also reported on the ‘control crime’ of financial fraud.^26^

**Table 1:** Reported outcome measures

Study	Design	Control	Study duration	Outcome categories
de Vocht *et al*, 2015^22^	CITS	Matched areas	6 years	Hospital admissions
de Vocht *et al*, 2017^26^	CITS	Financial fraud control crime	6 years	Alcohol crime
de Vocht *et al*, 2017^23^	CITS	Synthetic controls	6 years	Hospital admissionsViolent crimeAntisocial
Lock *et al*, 2017^20^	ITS	None	8 years	Ambulance
Pliakas *et al*, 2018^21^	ITS	None	8 years	AmbulanceAlcohol crimeAntisocial
de Vocht *et al*, 2020^24^	CITS	Synthetic controls	12 months	Emergency admissionsAmbulanceAlcohol crime
de Vocht *et al*, 2022^25^	ITS	Matched areas	7 years	Hospital admissionsAmbulanceCrime

CITScontrolled interrupted time seriesITS, interrupted time series

### Results of studies

#### Health outcomes

Three papers reported on hospital admissions (table 2). Two studies reported decreases in hospital admissions following the introduction of a local alcohol licensing policy intervention,^22 23^ in particular in relation to high-intensity policy,^22^ and one paper reported no significant change in emergency admission to hospital for alcohol.^24^ Three papers reported on ambulance call-out rates but changes were not statistically significant.^20 21 24^ de Vocht *et al*^25^ found no clear evidence of any associations between the involvement of public health teams in alcohol licensing and the public health or crime outcomes examined, nor between PHIAL scores and any outcomes.

**Table 2:** Summary of main results data

Author, yearlocation	Intervention	Results data
de Vocht *et al,*2017England^23^	Intervention areas were local areas with no specified CIZ and no rejection of new licensing applications in 2007/2008, but with both implemented in 2011/2012 and thereafter. Controls were local areas with neither policy	Alcohol-related hospital admissions reduced by 6.3% (95% credible intervals (CI) −12.8% to 0.2%, p=0.06). **Changing from ‘passive’ alcohol licensing intensity to ‘most intense’ on alcohol-related hospital admissions (average relative impact of −6.3% (95% CI −12.8% to 0.2%) over the 4-year period**).
		Violent crimes reduced by 4.6% to 2013 (95% CI −10.7% to 1.4%, p=0.13), and by 4.4% to 2015 (95% CI −13.7% to 4.9%, p=0.36)
		Sexual crimes—weak reduction up to 2013 (–8.4%, 95% CI −21.4% to 4.6%, p=0.20), and overall to 2015 (−4.6, 95% CI −18.1 to 8.9 0.50, p=0.50)
		Antisocial behaviour to 2013, −12.6% (95% CI −26.4 to 1.3, p=0.07. Overall reduction −14.3% (95% CI −32.9% to 44%, p=0.13)
de Vocht *et al*,2017England^26^	Degree of intensity of scrutiny/engagement in CIZ for each locality coded as ‘passive’, low, medium or highly activity (see de Vocht 2015^22^ for full description).	Alcohol-related violent crimes: Most ‘intense’ areas reduction from 6.1 per 1000 people in 2009 to 4.9 per 1000 people in 2013 (and back to 5.2 per 1000 people in 2014). ‘Passive’ areas on average reduction from 3.9/1000 in 2009 to 3.3/1000 in 2013 (and to 3.5/1000 in 2014).
		Alcohol-related sexual crimes: Most ‘intense’ areas reduced from 0.15 to 0.14 per 1000 people from 2009 until 2013. ‘Passive’ areas remained at ‘about’ 0.1 per 1000 people across this period.
		Public order offences: Passive areas registered rates reduced from 2.6 to 1.6 per 1000 people.Most ‘intense’ areas reduced from 4.6 to 2.9 per 1000 people.
Pliakas *et al,* 2018England^21^	Introducing a new alcohol licensing policy that included a comprehensive Cumulative Impact Policy (CIP) enforced in seven CIZs in one English Local Authority in 2013. Included a borough-wide guideline framework of closing times for businesses applying for new and variation alcohol licences including off-licences, nightclubs, restaurants, cafes and bars, hot food and drink from takeaways, and sales of alcohol to hotel residents.	Antisocial behaviour: CIZs (9.12%, 95% CI −9.21% to 31.14%); Non-CIZs (−0.67%, 95% CI −14.92% to 15.97%); local authority (4.25%, 95% CI −10.73% to 21.75%)
		Alcohol-related ambulance call-out rates: CIZ −2.50% (95% CI −12.74% to 8.95%); Non CIZ 8.83% (95% CI −5.51% to 25.35%); across the local authority 2.13% (95% CI −8.13% to 13.54%).
		Crime (overall): **CIZ −12.22%, (95% CI −17.95% to −6.09%); Non-CIZ −7.97%, (95% CI −13.96% to −1.56%); local authority: −10.32, (95% CI −15.19 to −5.18**).
de Vocht *et al,*2015England^22^	Examined the degree of intensity of scrutiny/engagement in CIZ: based on whether a licensing authority used CIZ (yes/no); and whether any licences for new premises were successfully challenged. Aggregated for each available year to obtain a three-level metric. This cumulative score was then divided into four categories: no activity (passive), and three levels of intensity (low, medium, high), based on tertiles of the distribution.	Alcohol-related hospital admissions: Medium intensity policy: 0.6% decrease annually.**High-intensity policy: 2% decrease in hospital admission rates (95% CI −3% to −2%) annually (p<0.05**).(Effect on slope: low −0.006 (SE 0.055); medium −0.065 (SE 0.058); high −0.229 (SE 0.067)
de Vocht *et al*, 2020England^24^	Three English local areas. Interventions consisted of: (1) the closure of a nightclub following reviews; (2) closure of a restaurant /nightclub following reviews and (3) implementation of new local licensing guidance (LLG).	Reported as: average impact at 12 months (95% CI) Posterior tail-area probability.Antisocial behaviour: case study 1 (nightclub closure): +8% (95% CI −63% to+49%) p=0.36; case study 2 (restaurant closure): −1% (95% CI −155% to −154%) p=0.48; case study 3 (new licensing guidance): −12% (95% CI −95% to+36%) p=0.40; post hoc evaluation of 4 month impact: **case study 1: −18% (95% CI −37% to −4%) p=0.01**
		Ambulance call-outs (all): case study 1: −9% (95% CI −36% to+20%) p=0.22
		Emergency admission to hospital for alcohol: case study 1: −19% (95% CI −155% to +113%) p=0.39
		Outcome: crime (all offences): case study 1: +4% (95% CI −69% to+77%), p=0.45. Case study 2: +0.3% (95% CI −61% to+49%) p=0.44
		Drunk and disorderly behaviour. Case study 3: −42% (95% CI −109% to +23%), p=0.10. Temporal falsification for drunk and disorderly behaviour 6 months earlier: −1% (95% CI −95% to +91%) p=0.49; 6 months later −27% (95% CI −115% to +61%), p=0.27. Spatial falsification for drunk and disorderly behaviour: Control area 1%–9% (95% CI −64% to +43%) p=0.36; control area 2%–53% (95% CI −119% to +14%) p=0.06; control area 3%–46% (95% CI −183% to +89%), p=0.23; control area 4 +27% (95% CI −106% to+156%), p=0.33; control area 5 +329% (95% CI −148% to+821%) p=0.08; control area 6 –64% (95% CI −196% to+60%), p=0.15.
		Sexual offences: case study 3: +5% (96% CI −95% to+90%), p=0.44
		Domestic violence: case study 3: +0.7% (95% CI −28% to+30%), p=0.48
de Vocht *et al*, 2012UK (England and Scotland)^25^	Measured public health engagement in the licensing system (not an action or decision of the licensing system itself) was investigated. ‘The Public Health engagement In Alcohol Licensing (PHIAL) Measure’ was developed, which included activities in six categories: staffing; reviewing licence applications; responding to licence applications; data usage; influencing licensing stakeholders or policy and public involvement.	Associations (per PHIAL unit exposure) of primary exposure metric (18 months average PHIAL score) and selected outcomes. Effect estimate describes the change in outcome (per 100 events) with one unit change in 18-month average PHIAL score. Adjusted outcome effect, 95% CI, p value.
		Hospital admissions: alcohol-related hospital admissions (narrow) 0.0006 (95% CI −0.0065 to 0.0078), p=0.866. Acute alcohol-related hospital admissions 0.0033 (95% CI −0.0058 to 0.0123), p=0.4766 months lagged: alcohol-related hospital admissions (narrow) 0.0003 (95% CI −0.0067 to 0.0073), p=0.935. Acute alcohol-related hospital admissions 0.0029 (95% CI −0.0062 to 0.0120), p=0.534
		Mortality: alcohol-related mortality 0.0016 (95% CI −0.0015 to 0.0047), p=0.315. Alcohol-specific mortality 0.0035 (95% CI −0.0032 to 0.0102), p=0.300.6 months lagged: alcohol-related 0.0004 (95% CI −0.0027 to 0.0034), p=0.813. Alcohol-specific mortality 0.0016 (95% CI −0.0050 to 0.0082), p=0.640
		Ambulance callouts (call): 0.0004 (95% CI −0.0016 to 0.0024), p=0.709. 6 months lagged: 0.0007 (95% CI −0.0014 to 0.0027), p=0.521
		Crime: public order offences 0.0074 (95% CI −0.0006 to 0.0153), p=0.068. Sexual crimes −0.0007 (95% CI −0.0055 to 0.0042), p=0.789. Violent crimes 0.0010 (95% CI −0.0025 to 0.0044), p=0.574. 6 months lagged: public order offences 0.0105 (95% CI 0.0027 to 0.0183), p=0.008. Sexual crimes −0.0026 (95% CI −0.0072 to 0.0021), p=0.280. Violent crimes 0.0003 (95% CI −0.0031 to 0.0037), p=0.853
Lock *et al,* 2017England^20^	The introduction of a new cumulative impact zone in the London borough of Islington.	Not provided—descriptive data only

Bold text: statistically significant change.

CIZCumulative Impact Zones

#### Well-being and crime outcomes

Three papers reported on antisocial behaviour. One paper reported temporary reductions in antisocial behaviour at 4 months which were not sustained.^21^ Two papers reported non-significant changes in antisocial behaviour.^23 24^ Changes in reporting of antisocial behaviour were noted as a limiting factor.^23^

Three papers reported on alcohol-related crime. Two papers reported reductions in alcohol-related violent crimes.^23 26^ Reported declines were time-limited, but greater in areas of more intense intervention.^26^ In addition, de Vocht *et al*^24^ reported small reductions in drunk and disorderly behaviour. Two papers also reported non-significant reductions in sexual crimes,^23 26^ again with the effect of more intense intervention being time-limited.^26^

Pliakas *et al*^21^ also reported that a statistically significant immediate drop in overall crime rates was reversed over the longer term. de Vocht *et al*^23^ reported non-significant reductions in violent crimes; and de Vocht *et al*^26^ reported that public order offences declined more steeply in high-intensity areas than areas with less intense policy.

## Discussion

We undertook a systematic review to identify and synthesise the current evidence base on the impact of local alcohol licensing decisions. Given the potential for these decisions to have a measurable impact on health and related outcomes in the local population, and the involvement of local public health teams in licensing decisions, it is important to understand the extent to which changes to local licensing policy may represent an effective public health intervention. We identified seven papers based on quantitative evaluation of local alcohol policy approaches.

The studies used quasi-experimental designs including natural experiments, some with local comparator areas and others employing the use of synthetic controls. In natural experiment studies, the exposure allocation is not controlled by researchers but is assumed to be ‘as-if randomised’.^27^ As these studies evaluate the impact of events or process that leads to differences in exposure, they are potentially less susceptible to bias than other observational study designs.^27^ However, the studies with synthetic controls are challenging to conduct over long time scales, with uncertainty around the synthetic measures increasing over time as these are static measures, and therefore, become less reliable over time. As naturally occurring populations also contain significant variance, the difficulties in identifying the most appropriate control populations also renders the use of naturally occurring comparison groups problematic. Although propensity scoring methods could be used to find ‘best matches’, synthetic controls may be particularly useful when suitable comparison groups are not available. There is a challenge over all in clear defining and differentiating the study designs employed.

Some limited changes in health and crime-related outcomes as a result of local intervention were noted but were often reversed over a longer timeframe and most changes were not significant overall. In terms of statistically significant changes in health outcomes, de Vocht *et al*^22^ reported that higher intensity local licensing policy (CIZ and increased licensing enforcement) was associated with a 2% decrease in hospital admission rates annually, and de Vocht *et al*^23^ reported an effect of changing from ‘passive’ alcohol licensing intensity to ‘most intense’ on alcohol-related hospital admissions. In addition, de Vocht *et al*^24^ reported a significant reduction in antisocial behaviour for one case study only, and Pliakas *et al*^21^ reported some significant reductions in overall crime.

Overall, there is very little evidence to directly link local decisions in alcohol licensing to consistent or sustained changes to health or crime outcomes downstream. This is disappointing given that complementary qualitative evidence indicates that local public health teams can provide valued input into alcohol licensing in ways which had been expected to facilitate reductions in alcohol harms.^28^ While the engagement of local authority public health teams in alcohol licensing decisions may influence those licensing decisions successfully, the changes may still not influence health outcomes due to the limited capacity (and therefore impact) of local decisions vs national policies.

It is important to consider the use of novel methods including synthetic control areas within the natural experiments undertaken. The method makes assumptions that the relationship between the control and the intervention areas is stable,^23^ which is unlikely in reality. However, confounders such as national policies or austerity that affect all time series, will automatically be controlled for.^23^ This, along with relatively large samples sizes, suggests that if there was an important effect these methods would identify it, and therefore, it is reasonable to conclude there is no evidence of an effect.

The mechanisms by which changes in alcohol availability may impact on alcohol-related harms are poorly examined and understood^11^ with greater clarity needed as to how changes in temporal and physical alcohol availability impact on alcohol consumption choices and patterns. Despite the involvement of stakeholders from all four of the UK home nations, the studies that we identified were all conducted in England, with the exception of de Vocht *et al*^25^ which also included data from Scotland. Given that alcohol policy approaches vary significantly in Wales, Scotland and especially Northern Ireland (with significantly different licensing systems in place), it is not possible to generalise from studies conducted in England to those nations.

Conversations with our stakeholders support the argument that public health practitioners with responsibility for alcohol licensing decisions can have only a limited positive impact in terms of encouraging the consideration of health outcomes and health data within decision-making. As noted by the study authors themselves, such involvement likely has benefits in shaping the licensing system to take account of health issues longer term, but it is unrealistic to expect this to directly impact on measurable outcomes in health, well-being or crime over the time frames implemented in these studies.^23^ With CIZ only able to impact on new licensing decisions, the influences which can be exerted will always be dwarfed by the impact of alcohol outlets which already exist. The impact of local licensing decisions is further limited by the boom in online alcohol sales and rapid doorstep deliveries.^23^ It may simply be that regardless of the extent of public health involvement, the impact of local licensing decisions is not substantial enough to lead to changes in harms of a detectable magnitude.

### Study limitations

Our systematic review design was limited by the time frame in which we were required, by our funders, to complete the review process, leading to a rapid review methodology being selected. However, the limited evidence base identified gave us more time to ensure that our searches were exhaustive (including grey literature sources) which, alongside the thorough application of both additional searching techniques and stakeholder consultation, reassures us that further evidence sources have not been missed. Our review focused on the UK evidence as it was commissioned by a UK funder. Our searches (including initial scoping searches without a UK filter applied) did, however, suggest that there was no significant volume of evidence from other countries. With more time and resources, potentially relevant further international evidence may have been identified, however, the particular context of the UK licensing arrangements would render it less relevant to our specific review question.

### Conclusions

We identified a small volume of evidence regarding the health (and related) impacts of alcohol licensing decision undertaken in local authorities. Despite relatively sophisticated study designs and some longer-term analysis, the evidence we identified did not demonstrate a consistent or sustained association between local decisions in alcohol licensing and health or crime outcomes downstream. It seems unlikely that the lack of measurable or consistent effects is purely due to the choice of study design or methods of analysis. Given that the impacts of local decisions are currently limited, greater regulatory powers are needed if local licensing interventions are to be an effective intervention to reduce alcohol-related harms. It is, therefore, unlikely that simply conducted more research of the type identified in this review (at least in England and Wales) would be beneficial, without first making regulatory changes to strengthening the impact of local decisions.

## supplementary material

10.1136/bmjph-2023-000533 online supplemental file 1

## Data Availability

Data sharing not applicable as no datasets generated and/or analysed for this study. All data relevant to the study are included in the article or uploaded as online supplemental information.
