# Supplementary material for: Evaluating the impact of local alcohol licensing decisions on outcomes for the community: a systematic review
Source: BMJ Public Health. 2024 Jan 18;2(1):e000533. doi: 10.1136/bmjph-2023-000533 (PMC11816718; doi:10.1136/bmjph-2023-000533)
Supplement: online supplemental file 1 [file bmjph-2-1-s001.pdf]

Supplementary material for paper

1. Sample search strategy

Ovid MEDLINE(R) and Epub Ahead of Print, In-Process, In-Data-Review & Other Non-Indexed Citations, Daily and Versions <1946 to October 05, 2022>

1 ((alcohol* or drink* or pub or pubs or bar or bars or nightclub* or night club*) adj3 (licen* or regulat* or law* or policy or policies)).mp. 5067

2 (alcohol adj2 (availab* or outlet* or density or proximity)).mp. 1502

3 exp Great Britain/ 386517

4 (national health service* or nhs*).ti,ab,in. 253386

5 (english not ((published or publication* or translat* or written or language* or speak* or literature or citation*) adj5 english)).ti,ab. 46314

6 (gb or "g.b." or britain* or (british* not "british columbia") or uk or "u.k." or united kingdom* or (england* not "new england") or northern ireland* or northern irish* or scotland* or scottish* or ((wales or "south wales") not "new south wales") or welsh*).ti,ab,jw,in. 2354884

7 (bath or "bath's" or ((birmingham not alabama*) or ("birmingham's" not alabama*) or bradford or "bradford's" or brighton or "brighton's" or bristol or "bristol's" or carlisle* or "carlisle's" or (cambridge not (massachusetts* or boston* or harvard*)) or ("cambridge's" not (massachusetts* or boston* or harvard*)) or (canterbury not zealand*) or ("canterbury's" not zealand*) or chelmsford or "chelmsford's" or chester or "chester's" or chichester or "chichester's" or coventry or "coventry's" or derby or "derby's" or (durham not (carolina* or nc)) or ("durham's" not (carolina* or nc)) or ely or "ely's" or exeter or "exeter's" or gloucester or "gloucester's" or hereford or "hereford's" or hull or "hull's" or lancaster or "lancaster's" or leeds* or leicester or "leicester's" or (lincoln not nebraska*) or ("lincoln's" not nebraska*) or (liverpool not (new south wales* or nsw)) or ("liverpool's" not (new south wales* or nsw)) or ((london not (ontario* or ont or toronto*)) or ("london's" not (ontario* or ont or toronto*)) or manchester or "manchester's" or (newcastle not (new south wales* or nsw)) or ("newcastle's" not (new south wales* or nsw)) or norwich or "norwich's" or nottingham or "nottingham's" or oxford or "oxford's" or peterborough or "peterborough's" or plymouth or "plymouth's" or portsmouth or "portsmouth's" or preston or "preston's" or ripon or "ripon's" or salford or "salford's" or salisbury or "salisbury's" or sheffield or "sheffield's" or southampton or "southampton's" or st albans or stoke or "stoke's" or sunderland or "sunderland's" or truro or "truro's" or wakefield or "wakefield's" or wells or westminster or "westminster's" or winchester or "winchester's" or wolverhampton or "wolverhampton's" or (worcester not (massachusetts* or boston* or harvard*)) or ("worcester's" not (massachusetts* or boston* or harvard*)) or (york not ("new york*" or ny or ontario* or ont or toronto*)) or ("york's" not ("new york*" or ny or ontario* or ont or toronto*))))).ti,ab,in. 1662578

8 (bangor or "bangor's" or cardiff or "cardiff's" or newport or "newport's" or st asaph or "st asaph's" or st davids or swansea or "swansea's").ti,ab,in. 66592

9 (aberdeen or "aberdeen's" or dundee or "dundee's" or edinburgh or "edinburgh's" or glasgow or "glasgow's" or inverness or (perth not australia*) or ("perth's" not australia*) or stirling or "stirling's").ti,ab,in. 245103

10 (armagh or "armagh's" or belfast or "belfast's" or lisburn or "lisburn's" or londonderry or "londonderry's" or derry or "derry's" or newry or "newry's").ti,ab,in. 31892

11 or/3-10 2956101

12 (exp africa/ or exp americas/ or exp antarctic regions/ or exp arctic regions/ or exp asia/ or expoceania/) not (exp great britain/ or europe/) 3062003

13 11 not 12 2814856

14 (1 or 2) and 13 751

15 limit 14 to yr="2002 -Current" 649

2. Quality appraisal of included papers

2.1. Appraisal of peer reviewed papers

Revisions to the CASP Cohort quality appraisal checklist were made as follows:

Question 1: replace “cohort” with “sample”.

Question 6: remove “of individuals”.

CASP Cohort adapted.

1. Did the study address a clearly focused issue?
2. Was the sample recruited in an acceptable way?
3. Was the exposure accurately measured to minimise bias?
4. Was the outcome accurately measure to minimise bias?
5. Have the authors identified all important confounding factors / taken account of them in the design and analysis?
6. Was the follow-up complete enough?
7. What are the results of the study?
8. How precise are the results?
9. Do you believe the results?
10. Can the results be applied to the local population?
11. Do the results of the study fit with other available evidence?
12. What are the implications of the study for practice?

First author / year	Design	N=	1	2	3	4	5	6	7	8	9	10	11	12	Notes
De Vocht 2015	Controlled ITS (matched areas) 6 years.	3 LTLAs	Y	Y	Y	Y	Y	Y	Alcohol related hospital admissions reduced	2% decrease in hospital admission rates (95% CI -3% to -2%) annually (p<0.05).	Y	Y	Y	More intense licensing policies may reduce alcohol-related hospital admissions.	Uncomplicated study design. Potential for impact.
De Vocht 2017a	Controlled ITS (matched areas) 6 years.	284 LTLAs	Y	Y	Y	Y	Y	Y	Alcohol related crime reduced	Reduction from 6.1 per 1000 people in 2009 to 4.9 per 1000 people in 2013.	Y	Y	Y	More intense licensing policies saw stronger decline in in rates of violent crimes, sexual crimes and public order offences	Uncomplicated study design. Potential for impact.
De Vocht 2017b	Controlled ITS (matched areas) 6 years.	5 intervention and 86 control areas	Y	Y	Y	Y	Y	Y	Hospital admissions Violent crime Antisocial behaviour reduced	e.g. Alcohol related hospital admissions reduced by 6.3% (95% credible intervals (CI) -12.8% to 0.2%, p=0.06)	Y	Y	Y	Moderate reductions in outcomes associated with local alcohol licensing policies.	Level of analysis may not match to that of the intervention.
Pliakas 2018	ITS (no control) 8 years.	One LTLA	Y	Y	Y	Y	Y	Y	Reduced: Ambulance call outs; Alcohol related crime; Antisocial behaviour	e.g. alcohol-related ambulance call-out rates: CIZ −2.50% (95% CI −12.74, 8.95).	Y	Y	Y	More intensive, sustained intervention necessary for long term benefits.	No control.
De Vocht 2020	CITS (Synthetic control) 12 months	3 LTLA	Y	Y	Y	Y	Y	Y	Emergency admissions, Ambulance call outs, Alcohol crime	e.g. Ambulance call outs: Case study 1: -9% (-36%, +20%) p=0.22	Y	Y	Y	Closure or restriction of alcohol venues and alcohol licensing may have a positive impact on health and crime.	Risk of implicit or explicit bias in the selection of the controls.

First author / year	Design	N=	1	2	3	4	5	6	7	8	9	10	11	12	Notes
De Vocht 2022	CITS (Matched areas)<br>18 months	39 Public Health Teams	Y	Y	Y	Y	Y	Y	Hospital admissions, Ambulance call outs<br>Alcohol crime	Adjusted outcome Effect, 95%Confidence Interval, P value<br>e.g. Acute alcohol-related hospital admissions 0.0033 -0.0058,0.0123 0.476	Y	Y	Y	No clear evidence that allocating PHT resources to alcohol licensing is associated with outcomes.	Longer term outcomes needed to see if any effect.

LTLA – Lower Tier Local Authority

2.2. 2.2 Critical appraisal of grey literature

Grey literature – AACODS checklist

1. Authority (Y, N, U) – consider:

Identifying who is responsible for the intellectual content.

- Individual author:
    - Associated with a reputable organisation?
    - Professional qualifications or considerable experience?
    - Produced/published other work (grey/black) in the field?
    - Recognised expert, identified in other sources?
    - Cited by others? (use Google Scholar as a quick check)
    - Higher degree student under “expert” supervision?
- Organisation or group:
    - Is the organisation reputable? (e.g. W.H.O)
    - Is the organisation an authority in the field?
- In all cases:
    - Does the item have a detailed reference list or bibliography?

2. Accuracy (Y, N, U) – consider:

- Does the item have a clearly stated aim or brief?

- Is so, is this met?
- Does it have a stated methodology?
- If so, is it adhered to?
- Has it been peer-reviewed?
- Has it been edited by a reputable authority?
- Supported by authoritative, documented references or credible sources?
- Is it representative of work in the field?
- If No, is it a valid counterbalance?
- Is any data collection explicit and appropriate for the research?
- if item is secondary material (e.g. a policy brief of a technical report) refer to the original. Is it an accurate, unbiased interpretation or analysis?

3. Coverage (Y, N, U) – consider:

All items have parameters which define their content coverage. These limits might mean that a work refers to a particular population group, or that it excluded certain types of publication. A report could be designed to answer a particular question, or be based on statistics from a particular survey.

- Are any limits clearly stated?

4. Objectivity (Y, N, U) – consider:

It is important to identify bias, particularly if it is unstated or unacknowledged.

- Opinion, expert or otherwise, is still opinion: is the author's standpoint clear?
- Does the work seem to be balanced in presentation?

5. Date (Y, N, U) – consider:

For the item to inform your research, it needs to have a date that confirms relevance

- Does the item have a clearly stated date related to content? No easily discernible date is a strong concern.
- If no date is given, but can be closely ascertained, is there a valid reason for its absence?
- Check the bibliography: have key contemporary material been included?

6. Significance (Y, N, U) – consider:

This is a value judgment of the item, in the context of the relevant research area

- Is the item meaningful? (this incorporates feasibility, utility and relevance)

- Does it add context?
- Does it enrich or add something unique to the research?
- Does it strengthen or refute a current position?
- Would the research area be lesser without it?
- Is it integral, representative, typical?
- Does it have impact? (in the sense of influencing the work or behaviour of others)

**First author / year**	**Design**	**N=**	**1**	**2**	**3**	**4**	**5**	**6**	**Summary**
Lock 2017	Evaluation report.<br>ITS (no control)<br>8 years.	One London borough	Y	Y	N	N	Y	N	Report. Reports on the same data as Pliakas 2018.

3. **Excluded full paper with reasons**

Paper	Reason
Anderson P, Baumberg B. Cost benefit analyses of alcohol policy - A primer. Cost benefit analyses of alcohol policy - A primer. 2010.	Exclude – not UK, not licensing
Anderson P, Jané-Llopis E, Hasan OSM, Rehm J. City-based action to reduce harmful alcohol use: review of reviews. F1000Research. 2018;7.	Systematic review. Checked references – non relevant
Angus C, Holmes J, Maheswaran R, Green MA, Meier P, Brennan A. Mapping patterns and trends in the spatial availability of alcohol using low-level geographic data: a case study in England 2003–2013. International journal of environmental research and public health. 2017;14(4):406.	Outlet density. Not local licensing.
Atkinson J-A, Prodan A, Livingston M, Knowles D, O'Donnell E, Room R, et al. Impacts of licensed premises trading hour policies on alcohol-related harms. Addiction. 2018;113(7):1244-51.	Exclude – not UK
Boshari, T., C. A. Sharpe, A. J. Poots, H. Watt, S. Rahman, and R. J. Pinder. 2020. 'Observational study of the association between diverse licensed premises types and alcohol-related violence in an inner-London borough', *Journal of Epidemiology & Community Health*, 74: 1016-22.	Outlet density
Brennan A, Meier P, Purshouse R, Rafia R, Meng Y, Hill-Macmanus D, et al. The Sheffield Alcohol Policy Model: A mathematical description. Health Economics. 2015;24(10):1368-88.	Exclude – methodology paper
Brennan A, Meng Y, Holmes J, Hill-McManus D, Meier PS. Potential benefits of minimum unit pricing for alcohol versus a ban on below cost selling in England 2014: modelling study. BMJ (Clinical research ed). 2014;349:g5452.	Exclude – minimum pricing
Bryden A, Roberts B, McKee M, Petticrew M. A systematic review of the influence on alcohol use of community level availability and marketing of alcohol. Health & place. 2012;18(2):349-57.	Systematic review. Checked references – non relevant
Burton R, Henn C, Lavoie D, O'Connor R, Perkins C, Sweeney K, et al. A rapid evidence review of the effectiveness and cost-effectiveness of alcohol control policies: an English perspective. Lancet (London, England). 2017;389(10078):1558-80.	Review – check references
Callan, C. M., and A. A. Boyle. 2018. 'Has the Licensing Act 2003 affected violence rates in England and Wales? A systematic review of hospital and police studies', *European Journal of Emergency Medicine*, 25: 304-11	Systematic review. Checked references – non relevant
Campbell CA, Hahn RA, Elder R, Brewer R, Chattopadhyay S, Fielding J, et al. The effectiveness of	Systematic review.

limiting alcohol outlet density as a means of reducing excessive alcohol consumption and alcohol-related harms. American journal of preventive medicine. 2009;37(6):556-69.	Checked references – non relevant
David, G., R. Cooper, S. Dixon, and J. Holmes. 2022. 'Exploring the implementation of public involvement in local alcohol availability policy: the case of alcohol licensing decision-making in England', *Addiction*, 117: 1163-72.	Qualitative
de Vocht F, Heron J, Mooney J, Angus C, Lock K, Egan M, et al. P66 Cross-sectional and longitudinal associations between alcohol licensing policies, outlet density and deprivation and population health and crime in England. BMJ Publishing Group Ltd; 2016.	Conference abstract
De Vocht F, Heron J, Mooney J, Brennan A, Lock K, Campbell R, et al. Estimating the measurable impact of local alcohol licensing policies on population health in England using ecological longitudinal data. The Lancet. 2015;386:S33.	Conference abstract
de Vocht F, McQuire C, Brennan A, Egan M, Angus C, Kaner E, et al. Evaluating the impact of individual alcohol licensing decisions on local health and crime: a natural experiment with synthetic controls. The Lancet. 2019;394:S35.	Conference abstract
de Vocht F, McQuire C, Ferraro C, Williams P, Henney M, Angus C, et al. 2021 Associations between public health team engagement in local alcohol licensing and public health and crime outcomes in England and Scotland: a timeseries analysis. The Lancet. 2021;398:S40	Conference abstract
de Vocht F, Tilling K, Campbell R, Hickman M. 2016 Inferring the intervention effect of local alcohol licensing policies on hospital admission and violent crime: a natural experiment with Bayesian synthetic controls. The Lancet. 2016;388:S43	Conference abstract
Deehan A. The prevention of alcohol-related crime: Operationalising situational and environmental strategies. Crime Prevention and Community Safety. 2004;6(1):43-52.	Review – check references
Drummond, D. C. 2000. 'UK government announces first major relaxation in the alcohol licensing laws for nearly a century: drinking in the UK goes 24-7', *Addiction*, 95: 997-8.	Discursive
Egan M, Brennan A, Buykx P, De Vocht F, Gavens L, Grace D, et al. Local policies to tackle a national problem: Comparative qualitative case studies of an English local authority alcohol availability intervention. Health & Place. 2016;41:11-8.	Qualitative
Egan M, Pliakas T. A MIXED METHODS EVALUATION OF A LOCAL-LEVEL ALCOHOL AVAILABILITY	Exclude - qualitative

INTERVENTION: REDUCING THE STRENGTH. Journal of Epidemiology and Community Health. 2015;69(Supplement 1):A70.	
El-Maaytah, M., S. F. Smith, W. Jerjes, T. Upile, A. Petrie, N. Kalavrezos, P. Ayliffe, L. Newman, C. Hopper, and T. Lloyd. 2008. 'The effect of the new "24 hour alcohol licensing law" on the incidence of facial trauma in London', *British Journal of Oral & Maxillofacial Surgery*, 46: 460-3.	National policy change
Fitzgerald N, Angus K, Emslie C, Shipton D, Bauld L. Gender differences in the impact of population-level alcohol policy interventions: evidence synthesis of systematic reviews. Addiction. 2016;111(10):1735-47.	Systematic review Check references
Fitzgerald N, Cairney P. National objectives, local policymaking: public health efforts to translate national legislation into local policy in Scottish alcohol licensing. Evidence & Policy. 2022:1-21.	Qualitative
Fitzgerald N, Egan M, De Vocht F, Angus C, Nicholls J, Shortt N, et al. Exploring the impact of public health teams on alcohol premises licensing in England and Scotland (ExILEnS): procotol for a mixed methods natural experiment evaluation. BMC medical research methodology. 2018;18(1):1-14.	Protocol
Fitzgerald N, Nicholls J, Winterbottom J, Katikireddi SV. Implementing a public health objective for alcohol premises licensing in Scotland: a qualitative study of strategies, values, and perceptions of evidence. International journal of environmental research and public health. 2017;14(3):221.	Qualitative
Fitzgerald N, Winterbottom J, Nicholls J. Democracy and power in alcohol premises licensing: a qualitative interview study of the Scottish public health objective. Drug and Alcohol Review. 2018;37(5):607-15.	Qualitative
Fone D, Morgan J, Fry R, Rodgers S, Orford S, Farewell D, et al. Change in alcohol outlet density and alcohol-related harm to population health (CHALICE): a comprehensive record-linked database study in Wales. Public Health Research. 2016;4(3):1-184.	Outlet density. Not local licensing.
Forsyth, A. J. 2008. 'Banning glassware from nightclubs in Glasgow (Scotland): observed impacts, compliance and patron's views', *Alcohol & Alcoholism*, 43: 111-7.	Not licensing
Foster, S., G. Trapp, P. Hooper, W. H. Oddy, L. Wood, and M. Knuiman. 2017. 'Liquor landscapes: Does access to alcohol outlets influence alcohol consumption in young adults?', *Health & Place*, 45: 17-23.	Alcohol consumption outcomes only
Gartner A, Farewell DM, Morgan J, Rodgers S, Orford S, Fry R, et al. Association between alcohol outlet density and alcohol-related mortality in Wales: An e-cohort study. The Lancet. 2017;390(SPEC.ISS 1):S14.	Exclude – outlet density

Gavens L, Holmes J, Buykx P, De Vocht F, Egan M, Grace D, et al. Processes of local alcohol policy-making in England: Does the theory of policy transfer provide useful insights into public health decision-making? Health & place. 2019;57:358-64	Qualitative
Gmel G, Holmes J, Studer J. Are alcohol outlet densities strongly associated with alcohol-related outcomes? A critical review of recent evidence. Drug and alcohol review. 2016;35(1):40-54.	Outlet density. Not local licensing.
Hadfield P, Measham F. After the Act Alcohol licensing and the administrative governance of crime. Criminology & Public Policy. 2010;9(1):69-76.	Exclude - discursive
Hahn RA, Kuzara JL, Elder R, Brewer R, Chattopadhyay S, Fielding J, et al. Effectiveness of policies restricting hours of alcohol sales in preventing excessive alcohol consumption and related harms. American journal of preventive medicine. 2010;39(6):590-604.	Not UK
Hector D, McGill E, Grace D, Egan M. Challenging, co-operating and splitting: A qualitative analysis of how the trade press responded to cumulative impact policies in England and Wales. Drugs: Education, Prevention & Policy. 2019;26(1):104-12.	Exclude - qualitative
Holmes J, Guo Y, Maheswaran R, Nicholls J, Meier PS, Brennan A. The impact of spatial and temporal availability of alcohol on its consumption and related harms: a critical review in the context of UK licensing policies. Drug and alcohol review. 2014;33(5):515-25.	Systematic review Check references
Humphreys DK, Eisner MP, Wiebe DJ. Evaluating the impact of flexible alcohol trading hours on violence: an interrupted time series analysis. PloS one. 2013;8(2):e55581.	Exclude – national policy
Humphreys DK, Eisner MP. Do flexible alcohol trading hours reduce violence? A theory-based natural experiment in alcohol policy. Social science & medicine (1982). 2014;102:1-9.	Exclude – national policy
Humphreys DK, Eisner MP. Evaluating a natural experiment in alcohol policy The Licensing Act (2003) and the requirement for attention to implementation. Criminology & Public Policy. 2010;9(1):41-67.	Exclude – national policy
Humphreys, D. K., and D. M. Smith. 2013. 'Alcohol licensing data: why is it an underused resource in public health?', *Health & Place*, 24: 110-4.	Discursive
Jones, L. A., and S. Goodacre. 2010. 'Effect of 24-h alcohol licensing on emergency departments: the South Yorkshire experience', *Emergency Medicine Journal*, 27: 688-91.	National policy change
Livingston M, Chikritzhs T, Room R. Changing the density of alcohol outlets to reduce alcohol-related problems. Drug and alcohol review. 2007;26(5):557-66.	Outlet density. Not local licensing.

Maheswaran R, Green MA, Strong M, Brindley P, Angus C, Holmes J. Alcohol outlet density and alcohol related hospital admissions in England: a national small-area level ecological study. Addiction. 2018;113(11):2051-9.	Outlet density. Not local licensing.
Martin, J. G., R. Giulianotti, C. Bandura, S. Morrow, K. Hunt, A. Bancroft, and R. I. Purves. 2022. 'Stakeholder views of current laws surrounding alcohol at UK football matches: Is it a case of using a "sledgehammer to crack a nut"?', *International Journal of Drug Policy*, 107: 103789.	Qualitative
Martineau F, Tyner E, Lorenc T, Petticrew M, Lock K. Population-level interventions to reduce alcohol-related harm: an overview of systematic reviews. Preventive medicine. 2013;57(4):278-96.	Review – check references
McGill E. Evaluating local-level interventions to address alcohol-related harms in England: the development and application of a complex systems perspective to process evaluations: London School of Hygiene & Tropical Medicine; 2021.	Qualitative
Miller, P., A. Curtis, D. Palmer, L. Busija, J. Tindall, N. Droste, K. Gillham, K. Coomber, and J. Wiggers. 2014. 'Changes in injury-related hospital emergency department presentations associated with the imposition of regulatory versus voluntary licensing conditions on licensed venues in two cities', *Drug & Alcohol Review*, 33: 314-22.	Not UK<br>Australia
Mistral W, Velleman R, Templeton L, Mastache C. Local action to prevent alcohol problems: Is the UK Community Alcohol Prevention Programme the best solution? International Journal of Drug Policy. 2006;17(4):278-84.	Review – check references
Mooney J, Sattar Z, de Vocht F, Smolar M, Nicholls J, Ling J. Assessing the feasibility of using health information in alcohol licensing decisions: a case study of seven English local authorities. The Lancet. 2016;388:S79.	Qualitative
Mooney JD, Holmes J, Gavens L, de Vocht F, Hickman M, Lock K, et al. Investigating local policy drivers for alcohol harm prevention: a comparative case study of two local authorities in England. BMC public health. 2017;17(1):1-9.	Qualitative
Moriarty, K. J., and I. T. Gilmore. 2006. 'Licensing Britain's alcohol epidemic', *Journal of Epidemiology & Community Health*, 60: 94.	Discursive
Nepal S, Kypri K, Tekelab T, Hodder RK, Attia J, Bagade T, et al. Effects of extensions and restrictions in alcohol trading hours on the incidence of assault and unintentional injury: systematic review. Journal of studies on alcohol and drugs. 2020;81(1):5-23.	Review<br>Check references

Newton, A., S. J. Sarker, G. S. Pahal, E. van den Bergh, and C. Young. 2007. 'Impact of the new UK licensing law on emergency hospital attendances: a cohort study', *Emergency Medicine Journal*, 24: 532-4.	National policy change
Nicholls J. Public health and alcohol licensing in the UK: challenges, opportunities, and implications for policy and practice. Contemporary Drug Problems. 2015;42(2):87-105.	Discursive paper
Nicholls, J. 2012. 'Alcohol licensing in Scotland: a historical overview', *Addiction*, 107: 1397-403.	Narrative review<br>Check references
Nicholls, J., R. O'Donnell, L. Mahon, N. Fitzgerald, and ILEnS consortium Ex. 2022. ''Give us the real tools to do our jobs': views of UK stakeholders on the role of a public health objective for alcohol licensing', *Public Health*, 211: 122-27.	Qualitative
O'Donnell R, Mohan A, Purves R, Maani N, Egan M, Fitzgerald N. Navigating different public health roles in alcohol premises licensing: a multi-stakeholder interview study. The Lancet. 2021;398:S14.	Qualitative
Pliakas T, Egan M, Gibbons J, Ashton C, Hart J, Lock K. Do cumulative impact zones reduce alcohol availability in UK high streets? Assessment of a natural experiment introducing a new licensing policy. The Lancet. 2016;388(SPEC.ISS 1):94.	Exclude – no health outcomes
Pliakas T, Lock K, Jones A, Aalders S, Egan M. Getting shops to voluntarily stop selling cheap, strong beers and ciders: a time-series analysis evaluating impacts on alcohol availability and purchasing. Journal of public health (Oxford, England). 2019;41(1):110-8.	Exclude – no health outcomes
Popova S, Giesbrecht N, Bekmuradov D, Patra J. Hours and days of sale and density of alcohol outlets: Impacts on alcohol consumption and damage: A systematic review. Alcohol and Alcoholism. 2009;44(5):500-16.	Review – check references
Reynolds J, Engen J, McGrath M, Pashmi G, Andrews M, Egan M, et al. Influences on public health contributions to alcohol licensing processes in local government in England: A mixed-methods study. The Lancet. 2017;390(SPEC.ISS 1):S77.	Exclude - qualitative
Reynolds, J. 2022. 'Framings of risk and responsibility in newsprint media coverage of alcohol licensing regulations during the COVID-19 pandemic in England', *Drug & Alcohol Review*, 06: 06.	Qualitative
Reynolds, J., M. McGrath, E. Halliday, M. Ogden, S. Hare, M. Smolar, L. Lafortune, K. Lock, J. Popay, P. Cook, and M. Egan. 2020. ''The opportunity to have their say'? Identifying mechanisms of community engagement in local alcohol decision-making', *International Journal of Drug Policy*, 85: 102909.	Qualitative
Reynolds, J., M. McGrath, J. Engen, G. Pashmi, M. Andrews, C. Sharpe, M. Egan, J. Lim, and K. Lock.	Qualitative

2019. ''A true partner around the table?' Perceptions of how to strengthen public health's contributions to the alcohol licensing process', *Journal of Public Health*, 41: e1-e8.	
Reynolds, J., M. McGrath, J. Engen, G. Pashmi, M. Andrews, J. Lim, and K. Lock. 2018. 'Processes, practices and influence: a mixed methods study of public health contributions to alcohol licensing in local government', *BMC Public Health*, 18: 1385.	Qualitative
Ritson, B. 2004. 'Alcohol licensing laws: proposals for changes in Scottish law', *Alcohol & Alcoholism*, 39: 2-7.	Discursive
Sharpe CA, Poots A, Watt H, Williamson C, Franklin D, Pinder RJ. An observational study to examine how cumulative impact zones influence alcohol availability from different types of licensed outlets in an inner London Borough. BMJ open. 2019;9(9):e027320.	Exclude – no health outcomes
Sharpe CA, Poots AJ, Watt H, Franklin D, Pinder RJ. Controlling alcohol availability through local policy: an observational study to evaluate Cumulative Impact Zones in a London borough. Journal of public health (Oxford, England). 2018;40(3):e260-e8.	Exclude – no health outcomes
Somerville L, Thom B, Herring R. Public health participation in alcohol licensing decisions in England: the importance of navigating "contested space". Drugs and Alcohol Today. 2020;20(4):323-35.	Exclude - qualitative
Stevely AK, de Vocht F, Neves RB, Holmes J, Meier PS. Evaluating the effects of the Licensing Act 2003 on the characteristics of drinking occasions in England and Wales: a theory of change-guided evaluation of a natural experiment. Addiction. 2021;116(9):2348-59.	National policy change – not local licensing
Sumpter C, McGill E, Dickie E, Champo E, Romeri E, Egan M. Reducing the Strength: a mixed methods evaluation of alcohol retailers' willingness to voluntarily reduce the availability of low cost, high strength beers and ciders in two UK local authorities. BMC public health. 2016;16:448.	Exclude – no health outcomes
Thompson, C., S. Milton, M. Egan, and K. Lock. 2018. 'Down the local: A qualitative case study of daytime drinking spaces in the London Borough of Islington', *International Journal of Drug Policy*, 52: 1-8.	Qualitative
Ure C, Burns EJ, Hargreaves SC, Hidajat M, Coffey M, de Vocht F, et al. How can communities influence alcohol licensing at a local level? Licensing officers' perspectives of the barriers and facilitators to sustaining engagement in a volunteer-led alcohol harm reduction approach. International Journal of Drug Policy. 2021;98:103412.	Review<br>Check references
Wright, A. 2019. 'Local Alcohol Policy Implementation in Scotland: Understanding the Role of Accountability within Licensing', *International Journal of*	Qualitative

Environmental Research & Public Health [Electronic Resource], 16: 28.	
Laura Garius, Bethany Ward, Kirsty Teague & Andromachi Tseloni (2020) Evaluating harm-reduction initiatives in a night-time economy and music festival context, International Journal of Comparative and Applied Criminal Justice, 44:4, 321-333, DOI: 10.1080/01924036.2020.1719530	Exclude - Not licensing
Ward BM, O'Sullivan B, Buykx P. Evaluation of a local government "shelter and van" intervention to improve safety and reduce alcohol-related harm. BMC Public Health. 2018 Dec 12;18(1):1370. doi: 10.1186/s12889-018-6245-4. PMID: 30541525; PMCID: PMC6292016.	Exclude – not UK

4. Excluded grey sources

Report	Relevance
Studying individual-level factors relating to changes in alcohol and other drug use, and seeking… | Alcohol Change UK	Exclude not local policy. MUP and behaviour change
Local alcohol treatment and recovery service commissioning practices and their perceived outcomes… | Alcohol Change UK	Exclude not local policy. Treatment services
Alcohol and domestic abuse in the context of COVID-19 restrictions | Alcohol Change UK	Exclude not local policy. Domestic violence
Alcohol Health Alliance interim research findings on alcohol labelling | Alcohol Change UK	Exclude not local policy. Alcohol labelling
Rapid evidence review: The role of alcohol in contributing to violence in intimate partner… | Alcohol Change UK	Exclude not local policy. Intimate partner violence
Alcohol outlet density and alcohol-related hospital admissions in England: a geographical analysis | Alcohol Change UK	Exclude not local policy. Outlet density observational
A new approach to measuring drinking cultures in Britain | Alcohol Change UK	Exclude not local policy. Typology of drinking occasions
Alcohol pricing and purchasing among heavy drinkers in Edinburgh and Glasgow | Alcohol Change UK	Exclude not local policy. Minimum pricing
One too many? Sales to drunk customers: policy, enforcement and responsibility | Alcohol Change UK	Exclude not local policy. Drunk customer restrictions
A national study of acute care Alcohol Health Workers | Alcohol Change UK	Exclude not local policy. Alcohol health workers
Using licensing to protect public health: from evidence to practice | Alcohol Change UK	Full paper Exclude qualitative
Evaluation of the impact of public health transfer to local authorities on alcohol | Alcohol Change UK	Full paper Exclude – not health outcomes
ADPH ADPH response to the introduction of a Vaccine Taskforce style approach to tackling health challenges - ADPH	Exclude not local policy. News item

ADPH Statement: responding to Government's 10 year drugs strategy - ADPH	Exclude not local policy. Drugs misuse
ADPH Statement: Response to the independent review of drugs - ADPH	Exclude not local policy. Drugs misuse
ADPH New research shows alcohol minimum unit price could save almost 8,000 lives in north of England - ADPH	Exclude not local policy. Minimum pricing. Discursive
ADPH ADPH responds to impact of Minimum Unit Pricing for alcohol in Scotland - ADPH	Exclude not local policy. Minimum pricing. Discursive.
ADPH ADPH Policy Positions - ADPH	Exclude not local policy. Background
ADPH House of Commons Health Committee session on MUP: ADPH Evidence (Jan 2018) - ADPH	Exclude not local policy. Minimum pricing.
ADPH ADPH Response to Public Health (Minimum Price for Alcohol) (Wales) Bill Consultation - ADPH	Exclude not local policy. Minutes of meeting
ADPH ADPH Statement on Supreme Court Ruling on Minimum Unit Pricing - ADPH	Exclude not local policy. Minimum pricing
ADPH ADPH Consultation Response: Alcohol Structures - ADPH	Exclude not local policy. Minimum pricing
ADPH ADPH statement: Public Health England Alcohol Harm Evidence Review - ADPH	Exclude not local policy. Minimim pricing
ADPH Licensing Act 2003 committee - ADPH Consultation Response - ADPH	Exclude not local policy. Licensing act consultation
ADPH ADPH Response to the Welsh Government Consultation on Draft Public Health (Minimum Price for Alcohol) (Wales) Bill - ADPH	Exclude not local policy. Minimum pricing
ADPH Joint Review - Public Health England and the Association of Directors of Public Health - ADPH	Full paper Exclude – not health outcomes
Perfect_Storm_FINAL-compressed_SMALL.pdf (balancenortheast.co.uk)	Exclude not local policy. Discursive
Public_Perceptions_Report_2019.pdf (balancenortheast.co.uk)	Exclude not local policy.

	Survey
Alcohol_Charter_Digital.pdf (balancenortheast.co.uk)	Exclude not local policy.<br>National strategy
2010 to 2015 government policy: harmful drinking - GOV.UK (www.gov.uk)	Full paper<br>Exclude – not health outcomes
Prevention of drug and alcohol dependence - GOV.UK (www.gov.uk)	Exclude not local policy.<br>Preventing dependence
Alcohol Strategy: government response to Health Select Committee - GOV.UK (www.gov.uk)	Exclude not local policy.<br>Meeting notes
Commissioning quality standard: alcohol and drug services - GOV.UK (www.gov.uk)	Exclude not local policy.<br>Treatment services
NDTMS: consent and privacy notice - GOV.UK (www.gov.uk)	Exclude not local policy.
Alcohol and drug misuse prevention and treatment guidance - GOV.UK (www.gov.uk)	Full paper – check all links<br>Exclude – no info on licensing effectiveness
Alcohol: applying All Our Health - GOV.UK (www.gov.uk)	Exclude not local policy.<br>Healthy choices
Local Alcohol Profiles for England (LAPE) - GOV.UK (www.gov.uk)	Exclude not local policy.<br>Alcohol data sources
Alcohol and drug misuse and treatment statistics - GOV.UK (www.gov.uk)	Exclude not local policy.<br>Alcohol data sources
Alcohol and drugs evidence reviews and inquiries - GOV.UK (www.gov.uk)	Exclude not local policy.<br>Alcohol data sources
https://publichealthscotland.scot/publications/an-analysis-plan-for-the-evaluation-of-the-impact-of-alcohol-minimum-unit-pricing-on-deaths-and-hospital-admissions-in-scotland	Exclude not local policy.<br>Minimum pricing protocol
https://publichealthscotland.scot/publications/mup-evaluation-evidence-synthesis-protocol	Exclude not local policy.<br>Minimum pricing protocol

https://publichealthscotland.scot/publications/evaluating-the-impact-of-minimum-unit-pricing-mup-on-sales-based-alcohol-consumption-in-scotland-at-three-years-post-implementation/	Exclude not local policy. Minimum pricing
https://publichealthscotland.scot/publications/evaluating-the-impact-of-mup-on-alcohol-products-and-prices-2022/	Exclude not local policy. Minimum pricing
https://publichealthscotland.scot/publications/trade-responses-to-restrictions-on-the-marketing-of-unhealthy-commodities/trade-responses-to-restrictions-on-the-marketing-of-unhealthy-commodities/	Exclude not local policy. Pricing and reformulating
https://publichealthscotland.scot/publications/national-drug-and-alcohol-treatment-waiting-times/national-drug-and-alcohol-treatment-waiting-times-1-april-2022-to-30-june-2022/	Exclude not local policy. Treatment waiting times
https://publichealthscotland.scot/publications/interim-monitoring-report-on-statutory-funded-residential-rehabilitation-placements/interim-monitoring-report-on-statutory-funded-residential-rehabilitation-placements-placements-approved-by-alcohol-and-drug-partnerships-between-01-april-2021-to-30-june-2022/	Exclude not local policy. Rehabilitation funding
https://publichealthscotland.scot/publications/supplementary-information-for-the-national-benchmarking-report-on-implementation-of-the-medication-assisted-treatment-mat-standards/supplementary-information-for-the-national-benchmarking-report-on-implementation-of-the-medication-assisted-treatment-mat-standards-202122/	Exclude not local policy. Treatment
https://publichealthscotland.scot/publications/interim-monitoring-report-on-statutory-funded-residential-rehabilitation-placements/interim-monitoring-report-on-statutory-funded-residential-rehabilitation-placements-placements-approved-by-alcohol-and-drug-partnerships-between-01-april-2021-to-31st-march-2022/	Exclude not local policy. Treatment
https://publichealthscotland.scot/publications/review-of-alcohol-marketing-restrictions-in-seven-european-countries/review-of-alcohol-marketing-restrictions-in-seven-european-countries-14-june-2022/	Full paper Exclude – case studies not UK
https://publichealthscotland.scot/publications/evaluating-the-impact-of-minimum-unit-pricing-in-scotland-on-people-who-are-drinking-at-harmful-levels/	Exclude not local policy. Minimum pricing
https://publichealthscotland.scot/publications/estimating-population-alcohol-consumption-in-scotland-the-impact-of-using-different-sources-of-alcohol-retail-sales-data/	Exclude not local policy. Data sources
https://publichealthscotland.scot/publications/estimating-population-alcohol-consumption-in-scotland-assessing-the-validity-and-reliability-of-alcohol-retail-sales-data/	Exclude not local policy. Data sources
https://publichealthscotland.scot/publications/evaluating-the-impact-of-minimum-unit-pricing-mup-of-alcohol-in-scotland-on-cross-border-purchasing/	Exclude not local policy. Minimum pricing
https://publichealthscotland.scot/publications/alcohol-sales-and-harm-in-scotland-during-the-covid-19-pandemic/	Exclude not local policy. Alcohol harms

https://publichealthscotland.scot/publications/evaluation-of-the-impact-of-alcohol-minimum-unit-pricing-mup-on-crime-and-disorder-public-safety-and-public-nuisance/	Exclude not local policy. Minimum pricing
https://publichealthscotland.scot/publications/impact-of-minimum-unit-pricing-among-people-who-are-alcohol-dependent-and-accessing-treatment-services-interim-report-structured-interview-data/	Exclude not local policy. Minimum pricing
https://publichealthscotland.scot/publications/evaluating-the-impact-of-minimum-unit-pricing-mup-on-the-price-distribution-of-off-trade-alcohol-in-scotland/	Exclude not local policy. Minimum pricing
https://publichealthscotland.scot/publications/evaluation-of-the-impact-of-mup-on-crime-and-disorder-public-safety-and-public-nuisance/	Exclude not local policy. Minimum pricing
https://publichealthscotland.scot/publications/evaluating-the-impact-of-mup-on-alcohol-products-and-prices/	Exclude not local policy. Minimum pricing
https://publichealthscotland.scot/publications/public-attitudes-to-minimum-unit-pricing-mup-for-alcohol-in-scotland/	Exclude not local policy. Minimum pricing
https://publichealthscotland.scot/publications/evaluating-the-impact-of-minimum-unit-pricing-mup-on-sales-based-alcohol-consumption-in-scotland-controlled-interrupted-time-series-analyses/	Exclude not local policy. Minimum pricing
https://publichealthscotland.scot/publications/evaluating-the-impact-of-alcohol-minimum-unit-pricing-mup-in-scotland-observational-study-of-small-retailers/	Exclude not local policy. Minimum pricing
https://publichealthscotland.scot/publications/practitioners-views-on-the-impact-of-mup-on-protecting-children-and-young-people/	Exclude not local policy. Minimum pricing
https://publichealthscotland.scot/publications/evaluating-the-impact-of-minimum-unit-pricing-in-scotland-small-convenience-store-study/	Exclude not local policy. Minimum pricing
https://www.publichealth.hscni.net/publications/focus-alcohol	Exclude not local policy. Alcohol units guide
https://www.publichealth.hscni.net/publications/damis-drug-and-alcohol-monitoring-and-information-system-activity-reports	Exclude not local policy. Alcohol use trends
https://www.publichealth.hscni.net/publications/drug-and-alcohol-procurement-stakeholder-engagement-report	Exclude not local policy. Treatment services
https://www.publichealth.hscni.net/publications/northern-ireland-alcohol-use-disorders-care-pathway-%E2%80%93-management-acute-hospital-setting	Exclude not local policy. Hospital care
https://www.publichealth.hscni.net/publications/alcohol-drugs-and-older-people	Exclude not local policy. Public help book

https://www.publichealth.hscni.net/publications/bdawg-scoping-report-drugs-and-alcohol-services-belfast	Full paper Exclude – not licensing
Alcohol licensing: a guide for public health teams - GOV.UK (www.gov.uk)	Full paper Exclude – guidance document no data on health outcomes
Alcohol licensing and public health - GOV.UK (www.gov.uk)	Full paper – check all case studies – contact for reports [see contacts below]
Alcohol licensing guidance - GOV.UK (www.gov.uk)	Exclude Links not relevant or already considered
Public health and alcohol licensing in England | Local Government Association	Duplicate
Home (alcohollicensing.org.uk)	Exclude No data
Local Alcohol Partnerships Group |	Exclude no data. Add to contacts list below
AFS response to Aberdeen overprovision consultation https://www.alcohol-focus-scotland.org.uk/media/310446/AFS-Aberdeen-City-Overprovision-Response-Feb-2018.pdf	Exclude – no data
Alcohol outlet availability and harm: https://www.alcohol-focus-scotland.org.uk/media/310762/alcohol-outlet-availability-and-harm-in-scotland.pdf	Exclude – not licensing policy
https://www.alcohol-focus-scotland.org.uk/media/310730/alcohol-outlet-availability-and-harm-in-aberdeen-city.pdf	Exclude – not licensing policy
Local licensing guide for forum members https://www.alcohol-focus-scotland.org.uk/media/440081/afs-local-licensing-guide-for-forum-members.pdf	Exclude – no data (how to make licensing policy)
MUP compliance (licensing) evaluation https://www.healthscotland.scot/media/2660/minimum-unit-pricing-for-alcohol-evaluation-compliance-study-english-july2019.pdf	Exclude – not licensing
https://www.alcohol-focus-scotland.org.uk/campaigns-policy/availability-and-licensing/	Exclude – no data
https://www.alcohol-focus-scotland.org.uk/media/310464/AFS-Angus-SLP-Suggestions-March-2018.pdf	Exclude – no data
https://www.alcohol-focus-scotland.org.uk/media/263071/AFS-Resource-Section-2.pdf	Exclude – no data
https://www.alcohol-focus-scotland.org.uk/media/440016/licensing-update.pdf	Exclude – no data

https://www.alcohol-focus-scotland.org.uk/media/263019/AFS-Impact-Report-2016-17.pdf	Exclude – no data
https://www.alcohol-focus-scotland.org.uk/media/174763/Event-summary-Dundee.pdf	Exclude – no data
https://www.alcohol-focus-scotland.org.uk/media/172202/Regional-licensing-event-summary-Glasgow.pdf	Exclude – no data
https://www.alcohol-focus-scotland.org.uk/media/173508/Regional-licensing-event-summary-Aberdeen.pdf	Exclude – no data
https://www.alcohol-focus-scotland.org.uk/media/310774/alcohol-availability-and-harm-briefing-may-18.pdf	Exclude – no data
https://www.alcohol-focus-scotland.org.uk/media/440022/resource-5-%E2%80%93-standard-operating-hours.pdf	Exclude – not effect of licensing (just licensing information)
https://www.alcohol-focus-scotland.org.uk/media/173050/AFS-licensing-publications.pdf	Exclude – no data
https://www.alcohol-focus-scotland.org.uk/media/114417/Four-Nations-report.pdf	Exclude – no data<br>Potentially relevant reference – added to list
https://www.alcohol-focus-scotland.org.uk/media/59902/Rethinking-alcohol-licensing.pdf	Exclude – no data
https://www.alcohol-focus-scotland.org.uk/media/95215/Using-licensing-to-protect-public-health.pdf	Exclude – not examining impact of local licensing
https://www.alcohol-focus-scotland.org.uk/media/185272/phe-alcohol-health-burden-report-2016.pdf	Exclude – no data
https://www.alcohol-focus-scotland.org.uk/news/scotlands-licensing-system-needs-clearer-direction/	Exclude – not quantitative
http://researchbriefings.files.parliament.uk/documents/CBP-7269/CBP-7269.pdf	Exclude – no data<br>Useful background on cumulative impact policies<br>Potentially relevant references – added to list
https://sphr.nihr.ac.uk/wp-content/uploads/2018/08/SPHR-final-report-Evaluating-the-impact-of-a-Cumulative-Impact-Zone.pdf	Full paper include<br>Data extracted
http://www.thelancet.com/pdfs/journals/lancet/PIIS0140-6736(16)32330-3.pdf	Exclude – no health outcomes<br>May be useful background (data on number

	of license applications granted in a CIZ)
https://www.ias.org.uk/2022/10/20/how-does-the-late-night-levy-spur-change-in-the-night-time-economy/	Exclude – not licensing
https://www.ias.org.uk/report/ias-response-to-call-for-evidence-and-views-on-the-licensing-and-registration-of-clubs-amendment-bill/	Exclude – no data
https://www.ias.org.uk/2020/05/13/evaluating-the-effect-of-individual-alcohol-licensing-decisions-on-local-health-and-crime/	Exclude - Blog post relating to included study
https://www.ias.org.uk/report/2019-ias-response-to-alcohol-and-late-night-refreshment-licensing-statistics-consultation/	Exclude – no data
https://www.ias.org.uk/2017/04/11/lords-licensing-act-needs-major-overhaul-but-problems-only-half-diagnosed-2/	Exclude – no data
The Licensing Act: its uses and abuses 10 years on https://www.ias.org.uk/uploads/pdf/IAS%20reports/rp22032016.pdf	Exclude – not quantitative
https://www.ias.org.uk/factsheet/availability/	Exclude – no data
https://www.ias.org.uk/wp-content/uploads/2020/12/Licensing-in-practice-%E2%80%93-the-availability-of-alcohol-in-UK-society.pdf	Exclude – no data
https://www.ias.org.uk/2017/06/02/anytime-anyplace-anywhere-comparing-policies-that-regulate-physical-availability-of-alcohol-in-australia-and-the-uk/	Exclude – no data

Report	Relevance
Studying individual-level factors relating to changes in alcohol and other drug use, and seeking… | Alcohol Change UK	Exclude not local policy. MUP and behaviour change
Local alcohol treatment and recovery service commissioning practices and their perceived outcomes… | Alcohol Change UK	Exclude not local policy. Treatment services
Alcohol and domestic abuse in the context of COVID-19 restrictions | Alcohol Change UK	Exclude not local policy. Domestic violence
Alcohol Health Alliance interim research findings on alcohol labelling | Alcohol Change UK	Exclude not local policy. Alcohol labelling
Rapid evidence review: The role of alcohol in contributing to violence in intimate partner… | Alcohol Change UK	Exclude not local policy. Intimate partner violence
Alcohol outlet density and alcohol-related hospital admissions in England: a geographical analysis | Alcohol Change UK	Exclude not local policy. Outlet density observational
A new approach to measuring drinking cultures in Britain | Alcohol Change UK	Exclude not local policy. Typology of drinking occasions
Alcohol pricing and purchasing among heavy drinkers in Edinburgh and Glasgow | Alcohol Change UK	Exclude not local policy. Minimum pricing
One too many? Sales to drunk customers: policy, enforcement and responsibility | Alcohol Change UK	Exclude not local policy. Drunk customer restrictions
A national study of acute care Alcohol Health Workers | Alcohol Change UK	Exclude not local policy. Alcohol health workers
Using licensing to protect public health: from evidence to practice | Alcohol Change UK	Full paper Exclude qualitative
Evaluation of the impact of public health transfer to local authorities on alcohol | Alcohol Change UK	Full paper Exclude – not health outcomes
ADPH ADPH response to the introduction of a Vaccine Taskforce style approach to tackling health challenges - ADPH	Exclude not local policy. News item

ADPH Statement: responding to Government's 10 year drugs strategy - ADPH	Exclude not local policy. Drugs misuse
ADPH Statement: Response to the independent review of drugs - ADPH	Exclude not local policy. Drugs misuse
ADPH New research shows alcohol minimum unit price could save almost 8,000 lives in north of England - ADPH	Exclude not local policy. Minimum pricing. Discursive
ADPH ADPH responds to impact of Minimum Unit Pricing for alcohol in Scotland - ADPH	Exclude not local policy. Minimum pricing. Discursive.
ADPH ADPH Policy Positions - ADPH	Exclude not local policy. Background
ADPH House of Commons Health Committee session on MUP: ADPH Evidence (Jan 2018) - ADPH	Exclude not local policy. Minimum pricing.
ADPH ADPH Response to Public Health (Minimum Price for Alcohol) (Wales) Bill Consultation - ADPH	Exclude not local policy. Minutes of meeting
ADPH ADPH Statement on Supreme Court Ruling on Minimum Unit Pricing - ADPH	Exclude not local policy. Minimum pricing
ADPH ADPH Consultation Response: Alcohol Structures - ADPH	Exclude not local policy. Minimum pricing
ADPH ADPH statement: Public Health England Alcohol Harm Evidence Review - ADPH	Exclude not local policy. Minimim pricing
ADPH Licensing Act 2003 committee - ADPH Consultation Response - ADPH	Exclude not local policy. Licensing act consultation
ADPH ADPH Response to the Welsh Government Consultation on Draft Public Health (Minimum Price for Alcohol) (Wales) Bill - ADPH	Exclude not local policy. Minimum pricing
ADPH Joint Review - Public Health England and the Association of Directors of Public Health - ADPH	Full paper Exclude – not health outcomes
Perfect_Storm_FINAL-compressed_SMALL.pdf (balancenortheast.co.uk)	Exclude not local policy. Discursive
Public_Perceptions_Report_2019.pdf (balancenortheast.co.uk)	Exclude not local policy.

	Survey
Alcohol_Charter_Digital.pdf (balancenortheast.co.uk)	Exclude not local policy. National strategy
2010 to 2015 government policy: harmful drinking - GOV.UK (www.gov.uk)	Full paper Exclude – not health outcomes
Prevention of drug and alcohol dependence - GOV.UK (www.gov.uk)	Exclude not local policy. Preventing dependence
Alcohol Strategy: government response to Health Select Committee - GOV.UK (www.gov.uk)	Exclude not local policy. Meeting notes
Commissioning quality standard: alcohol and drug services - GOV.UK (www.gov.uk)	Exclude not local policy. Treatment services
NDTMS: consent and privacy notice - GOV.UK (www.gov.uk)	Exclude not local policy.
Alcohol and drug misuse prevention and treatment guidance - GOV.UK (www.gov.uk)	Full paper – check all links Exclude – no info on licensing effectiveness
Alcohol: applying All Our Health - GOV.UK (www.gov.uk)	Exclude not local policy. Healthy choices
Local Alcohol Profiles for England (LAPE) - GOV.UK (www.gov.uk)	Exclude not local policy. Alcohol data sources
Alcohol and drug misuse and treatment statistics - GOV.UK (www.gov.uk)	Exclude not local policy. Alcohol data sources
Alcohol and drugs evidence reviews and inquiries - GOV.UK (www.gov.uk)	Exclude not local policy. Alcohol data sources
https://publichealthscotland.scot/publications/an-analysis-plan-for-the-evaluation-of-the-impact-of-alcohol-minimum-unit-pricing-on-deaths-and-hospital-admissions-in-scotland	Exclude not local policy. Minimum pricing protocol
https://publichealthscotland.scot/publications/mup-evaluation-evidence-synthesis-protocol	Exclude not local policy. Minimum pricing protocol

https://publichealthscotland.scot/publications/evaluating-the-impact-of-minimum-unit-pricing-mup-on-sales-based-alcohol-consumption-in-scotland-at-three-years-post-implementation/	Exclude not local policy. Minimum pricing
https://publichealthscotland.scot/publications/evaluating-the-impact-of-mup-on-alcohol-products-and-prices-2022/	Exclude not local policy. Minimum pricing
https://publichealthscotland.scot/publications/trade-responses-to-restrictions-on-the-marketing-of-unhealthy-commodities/trade-responses-to-restrictions-on-the-marketing-of-unhealthy-commodities/	Exclude not local policy. Pricing and reformulating
https://publichealthscotland.scot/publications/national-drug-and-alcohol-treatment-waiting-times/national-drug-and-alcohol-treatment-waiting-times-1-april-2022-to-30-june-2022/	Exclude not local policy. Treatment waiting times
https://publichealthscotland.scot/publications/interim-monitoring-report-on-statutory-funded-residential-rehabilitation-placements/interim-monitoring-report-on-statutory-funded-residential-rehabilitation-placements-placements-approved-by-alcohol-and-drug-partnerships-between-01-april-2021-to-30-june-2022/	Exclude not local policy. Rehabilitation funding
https://publichealthscotland.scot/publications/supplementary-information-for-the-national-benchmarking-report-on-implementation-of-the-medication-assisted-treatment-mat-standards/supplementary-information-for-the-national-benchmarking-report-on-implementation-of-the-medication-assisted-treatment-mat-standards-202122/	Exclude not local policy. Treatment
https://publichealthscotland.scot/publications/interim-monitoring-report-on-statutory-funded-residential-rehabilitation-placements/interim-monitoring-report-on-statutory-funded-residential-rehabilitation-placements-placements-approved-by-alcohol-and-drug-partnerships-between-01-april-2021-to-31st-march-2022/	Exclude not local policy. Treatment
https://publichealthscotland.scot/publications/review-of-alcohol-marketing-restrictions-in-seven-european-countries/review-of-alcohol-marketing-restrictions-in-seven-european-countries-14-june-2022/	Full paper Exclude – case studies not UK
https://publichealthscotland.scot/publications/evaluating-the-impact-of-minimum-unit-pricing-in-scotland-on-people-who-are-drinking-at-harmful-levels/	Exclude not local policy. Minimum pricing
https://publichealthscotland.scot/publications/estimating-population-alcohol-consumption-in-scotland-the-impact-of-using-different-sources-of-alcohol-retail-sales-data/	Exclude not local policy. Data sources
https://publichealthscotland.scot/publications/estimating-population-alcohol-consumption-in-scotland-assessing-the-validity-and-reliability-of-alcohol-retail-sales-data/	Exclude not local policy. Data sources
https://publichealthscotland.scot/publications/evaluating-the-impact-of-minimum-unit-pricing-mup-of-alcohol-in-scotland-on-cross-border-purchasing/	Exclude not local policy. Minimum pricing
https://publichealthscotland.scot/publications/alcohol-sales-and-harm-in-scotland-during-the-covid-19-pandemic/	Exclude not local policy. Alcohol harms

https://publichealthscotland.scot/publications/evaluation-of-the-impact-of-alcohol-minimum-unit-pricing-mup-on-crime-and-disorder-public-safety-and-public-nuisance/	Exclude not local policy. Minimum pricing
https://publichealthscotland.scot/publications/impact-of-minimum-unit-pricing-among-people-who-are-alcohol-dependent-and-accessing-treatment-services-interim-report-structured-interview-data/	Exclude not local policy. Minimum pricing
https://publichealthscotland.scot/publications/evaluating-the-impact-of-minimum-unit-pricing-mup-on-the-price-distribution-of-off-trade-alcohol-in-scotland/	Exclude not local policy. Minimum pricing
https://publichealthscotland.scot/publications/evaluation-of-the-impact-of-mup-on-crime-and-disorder-public-safety-and-public-nuisance/	Exclude not local policy. Minimum pricing
https://publichealthscotland.scot/publications/evaluating-the-impact-of-mup-on-alcohol-products-and-prices/	Exclude not local policy. Minimum pricing
https://publichealthscotland.scot/publications/public-attitudes-to-minimum-unit-pricing-mup-for-alcohol-in-scotland/	Exclude not local policy. Minimum pricing
https://publichealthscotland.scot/publications/evaluating-the-impact-of-minimum-unit-pricing-mup-on-sales-based-alcohol-consumption-in-scotland-controlled-interrupted-time-series-analyses/	Exclude not local policy. Minimum pricing
https://publichealthscotland.scot/publications/evaluating-the-impact-of-alcohol-minimum-unit-pricing-mup-in-scotland-observational-study-of-small-retailers/	Exclude not local policy. Minimum pricing
https://publichealthscotland.scot/publications/practitioners-views-on-the-impact-of-mup-on-protecting-children-and-young-people/	Exclude not local policy. Minimum pricing
https://publichealthscotland.scot/publications/evaluating-the-impact-of-minimum-unit-pricing-in-scotland-small-convenience-store-study/	Exclude not local policy. Minimum pricing
https://www.publichealth.hscni.net/publications/focus-alcohol	Exclude not local policy. Alcohol units guide
https://www.publichealth.hscni.net/publications/damis-drug-and-alcohol-monitoring-and-information-system-activity-reports	Exclude not local policy. Alcohol use trends
https://www.publichealth.hscni.net/publications/drug-and-alcohol-procurement-stakeholder-engagement-report	Exclude not local policy. Treatment services
https://www.publichealth.hscni.net/publications/northern-ireland-alcohol-use-disorders-care-pathway-%E2%80%93-management-acute-hospital-setting	Exclude not local policy. Hospital care
https://www.publichealth.hscni.net/publications/alcohol-drugs-and-older-people	Exclude not local policy. Public help book

https://www.publichealth.hscni.net/publications/bdawg-scoping-report-drugs-and-alcohol-services-belfast	Full paper Exclude – not licensing
Alcohol licensing: a guide for public health teams - GOV.UK (www.gov.uk)	Full paper Exclude – guidance document no data on health outcomes
Alcohol licensing and public health - GOV.UK (www.gov.uk)	Full paper – check all case studies – contact for reports [see contacts below]
Alcohol licensing guidance - GOV.UK (www.gov.uk)	Exclude Links not relevant or already considered
Public health and alcohol licensing in England | Local Government Association	Duplicate
Home (alcohollicensing.org.uk)	Exclude No data
Local Alcohol Partnerships Group |	Exclude no data. Add to contacts list below
AFS response to Aberdeen overprovision consultation https://www.alcohol-focus-scotland.org.uk/media/310446/AFS-Aberdeen-City-Overprovision-Response-Feb-2018.pdf	Exclude – no data
Alcohol outlet availability and harm: https://www.alcohol-focus-scotland.org.uk/media/310762/alcohol-outlet-availability-and-harm-in-scotland.pdf	Exclude – not licensing policy
https://www.alcohol-focus-scotland.org.uk/media/310730/alcohol-outlet-availability-and-harm-in-aberdeen-city.pdf	Exclude – not licensing policy
Local licensing guide for forum members https://www.alcohol-focus-scotland.org.uk/media/440081/afs-local-licensing-guide-for-forum-members.pdf	Exclude – no data (how to make licensing policy)
MUP compliance (licensing) evaluation https://www.healthscotland.scot/media/2660/minimum-unit-pricing-for-alcohol-evaluation-compliance-study-english-july2019.pdf	Exclude – not licensing
https://www.alcohol-focus-scotland.org.uk/campaigns-policy/availability-and-licensing/	Exclude – no data
https://www.alcohol-focus-scotland.org.uk/media/310464/AFS-Angus-SLP-Suggestions-March-2018.pdf	Exclude – no data
https://www.alcohol-focus-scotland.org.uk/media/263071/AFS-Resource-Section-2.pdf	Exclude – no data
https://www.alcohol-focus-scotland.org.uk/media/440016/licensing-update.pdf	Exclude – no data

https://www.alcohol-focus-scotland.org.uk/media/263019/AFS-Impact-Report-2016-17.pdf	Exclude – no data
https://www.alcohol-focus-scotland.org.uk/media/174763/Event-summary-Dundee.pdf	Exclude – no data
https://www.alcohol-focus-scotland.org.uk/media/172202/Regional-licensing-event-summary-Glasgow.pdf	Exclude – no data
https://www.alcohol-focus-scotland.org.uk/media/173508/Regional-licensing-event-summary-Aberdeen.pdf	Exclude – no data
https://www.alcohol-focus-scotland.org.uk/media/310774/alcohol-availability-and-harm-briefing-may-18.pdf	Exclude – no data
https://www.alcohol-focus-scotland.org.uk/media/440022/resource-5-%E2%80%93-standard-operating-hours.pdf	Exclude – not effect of licensing (just licensing information)
https://www.alcohol-focus-scotland.org.uk/media/173050/AFS-licensing-publications.pdf	Exclude – no data
https://www.alcohol-focus-scotland.org.uk/media/114417/Four-Nations-report.pdf	Exclude – no data<br>Potentially relevant reference – added to list
https://www.alcohol-focus-scotland.org.uk/media/59902/Rethinking-alcohol-licensing.pdf	Exclude – no data
https://www.alcohol-focus-scotland.org.uk/media/95215/Using-licensing-to-protect-public-health.pdf	Exclude – not examining impact of local licensing
https://www.alcohol-focus-scotland.org.uk/media/185272/phe-alcohol-health-burden-report-2016.pdf	Exclude – no data
https://www.alcohol-focus-scotland.org.uk/news/scotlands-licensing-system-needs-clearer-direction/	Exclude – not quantitative
http://researchbriefings.files.parliament.uk/documents/CBP-7269/CBP-7269.pdf	Exclude – no data<br>Useful background on cumulative impact policies<br>Potentially relevant references – added to list
https://sphr.nihr.ac.uk/wp-content/uploads/2018/08/SPHR-final-report-Evaluating-the-impact-of-a-Cumulative-Impact-Zone.pdf	Full paper include<br>Data extracted
http://www.thelancet.com/pdfs/journals/lancet/PIIS0140-6736(16)32330-3.pdf	Exclude – no health outcomes<br>May be useful background (data on number

	of license applications granted in a CIZ)
https://www.ias.org.uk/2022/10/20/how-does-the-late-night-levy-spur-change-in-the-night-time-economy/	Exclude – not licensing
https://www.ias.org.uk/report/ias-response-to-call-for-evidence-and-views-on-the-licensing-and-registration-of-clubs-amendment-bill/	Exclude – no data
https://www.ias.org.uk/2020/05/13/evaluating-the-effect-of-individual-alcohol-licensing-decisions-on-local-health-and-crime/	Exclude - Blog post relating to included study
https://www.ias.org.uk/report/2019-ias-response-to-alcohol-and-late-night-refreshment-licensing-statistics-consultation/	Exclude – no data
https://www.ias.org.uk/2017/04/11/lords-licensing-act-needs-major-overhaul-but-problems-only-half-diagnosed-2/	Exclude – no data
The Licensing Act: its uses and abuses 10 years on https://www.ias.org.uk/uploads/pdf/IAS%20reports/rp22032016.pdf	Exclude – not quantitative
https://www.ias.org.uk/factsheet/availability/	Exclude – no data
https://www.ias.org.uk/wp-content/uploads/2020/12/Licensing-in-practice-%E2%80%93-the-availability-of-alcohol-in-UK-society.pdf	Exclude – no data
https://www.ias.org.uk/2017/06/02/anytime-anyplace-anywhere-comparing-policies-that-regulate-physical-availability-of-alcohol-in-australia-and-the-uk/	Exclude – no data

Selected websites listed at end of Alcohol Charter report

**Name of organisation**	**Terms entered**	**Possible results (if any)**
Blenheim CDP	n/a	None – website down
Action Addiction	n/a	No search function. No obvious relevant material linked from home page.
With You (formerly AddAction)	n/a	No search function.
Adfam	Licensing	Response to Government consultation on MUP and licensing: https://adfam.org.uk/files/docs/adfam_alcoholresponse.pdf
Alcohol Focus Scotland	**Licensing**	AFS response to Aberdeen overprovision consultation https://www.alcohol-focus-scotland.org.uk/media/310446/AFS-Aberdeen-City-Overprovision-Response-Feb-2018.pdf<br>Alcohol outlet availability and harm: https://www.alcohol-focus-scotland.org.uk/media/310762/alcohol-outlet-availability-and-harm-in-scotland.pdf<br>and<br>https://www.alcohol-focus-scotland.org.uk/media/310730/alcohol-outlet-availability-and-harm-in-aberdeen-city.pdf<br>Local licensing guide for forum members https://www.alcohol-focus-scotland.org.uk/media/440081/afs-local-licensing-guide-for-forum-members.pdf<br>MUP compliance (licensing) evaluation

		https://www.healthscotland.scot/media/2660/minimum-unit-pricing-for-alcohol-evaluation-compliance-study-english-july2019.pdf
Addiction Professionals	"Licensing" "licensed"	Nothing found
Alcohol Health Alliance	No search – browsed resources	IAS - What publicans think about policy, public health and the changing trade https://www.ias.org.uk/uploads/pdf/IAS%20reports/rp26092017.pdf<br>"It's everywhere" report of the Commission on Alcohol Harm (2020): https://ahauk.org/resource/commission-on-alcohol-harm-report/
Balance (whatstheharm.co.uk)	No search	Nothing found
British Association for the Study of the Liver (BASL)	No search	Nothing found
Changing Lives	No search	Nothing found
Collective Voice	No search	Nothing found
Homeless Link	"Alcohol"	Nothing relevant found
Institute of Alcohol Studies	"Licensing"	https://www.ias.org.uk/2022/10/20/how-does-the-late-night-levy-spur-change-in-the-night-time-economy/<br><br>https://www.ias.org.uk/report/ias-response-to-call-for-evidence-and-views-on-the-licensing-and-registration-of-clubs-amendment-bill/<br><br>https://www.ias.org.uk/2020/05/13/evaluating-the-effect-of-individual-alcohol-licensing-decisions-on-local-health-and-crime/<br><br>https://www.ias.org.uk/report/2019-ias-response-to-alcohol-and-late-night-refreshment-licensing-statistics-consultation/<br><br>https://www.ias.org.uk/2017/04/11/lords-licensing-act-needs-major-overhaul-but-problems-only-half-diagnosed-2/<br><br>The Licensing Act: its uses and abuses 10 years on https://www.ias.org.uk/uploads/pdf/IAS%20reports/rp22032016.pdf
Medical Council on Alcohol	Licensing	Nothing found
Scottish Families affected by Alcohol and Drugs	Licensing	https://www.sfad.org.uk/engaging-with-the-licensing-process-a-blog-by-john-holleran
Scottish Health Action on Alcohol Problems (SHAAP)	Licensing	Mainly responses to Govt consultations on licensing policy but also two older reports:<br><br>https://www.shaap.org.uk/downloads/60-re-thinking-alcohol-licensing-september-2011-pdf/viewdocument/60.html

		https://www.shaap.org.uk/downloads/53-licensing-for-public-health-pdf/viewdocument/53.html (2009)
